# Microbiome Transmission During Sexual Intercourse Appears Stochastic and Supports the Red Queen Hypothesis

**DOI:** 10.3389/fmicb.2021.789983

**Published:** 2022-03-08

**Authors:** Zhanshan (Sam) Ma

**Affiliations:** ^1^Computational Biology and Medical Ecology Lab, State Key Laboratory of Genetic Resources and Evolution, Kunming Institute of Zoology, Chinese Academy of Sciences (CAS), Kunming, China; ^2^Center for Excellence in Animal Evolution and Genetics, Chinese Academy of Sciences (CAS), Kunming, China

**Keywords:** microbiome transmission, semen microbiome, vaginal microbiome, neutral theory, multi-site neutral model (MSN), red queen hypothesis, coevolution

## Abstract

Microbes inhabit virtually everywhere on and/or in our bodies, including the seminal and vaginal fluids. They have significant importance in maintaining reproductive health and protecting hosts from diseases. The exchange of microbes during sexual intercourse is one of the most direct and significant microbial transmissions between men and women. Nevertheless, the mechanism of this microbial transmission was little known. Is the transmission mode stochastic, passive diffusion similar to the random walk of particles, or driven by some deterministic forces? What is the microbial transmission probability? What are the possible evolutionary implications, particularly from the perspective of sexual reproduction (selection)? We tackle these intriguing questions by leveraging the power of Hubbell’s unified neutral theory of biodiversity, specifically implemented as the HDP-MSN (hierarchical Dirichlet process approximated multi-site neutral model), which allows for constructing truly multi-site metacommunity models, *simultaneously* including vaginal and semen microbiomes. By reanalyzing the microbiome datasets of seminal and vaginal fluids from 23 couples both before and after sexual intercourses originally reported by Mändar and colleagues, we found that the microbial transmission between seminal and vaginal fluids is a stochastic, passive diffusion similar to the random walk of particles in physics, rather than driven by deterministic forces. The transmission probability through sexual intercourse seems to be approximately 0.05. Inspired by the results from the HDP-MSN model, we further conjecture that the stochastic drifts of microbiome transmissions during sexual intercourses can be responsible for the homogeneity between semen and vaginal microbiomes first identified in a previous study, which should be helpful for sexual reproduction by facilitating the sperm movement/survival and/or egg fertilization. This inference seems to be consistent with the classic Red Queen hypothesis, which, when extended to the co-evolutionary interactions between humans and their symbiotic microbiomes, would predict that the reproductive system microbiomes should support sexual reproduction.

## Introduction

Microbes inhabit virtually every corner of our body, including semen, male and female genital tracts. The genital microbiome have great importance in maintaining reproductive health and protecting hosts from disease (Ravel et al., 2011; Gajer et al., 2012; Macklaim et al., 2013). Studies show that the dysbiosis of vaginal microbiota is closely linked to an increased risk of certain diseases, such as bacterial vaginosis (BV) and sexually transmitted infections (e.g., Ma et al., 2012; Lewis et al., 2017; Smith and Ravel, 2017; van de Wijgert, 2017). Although the microbiome in the male genital tract exists primarily in the urethra and coronary sulcus, researchers typically use semen to study the microbiome of the male genital tract. Semen microbiome has been found to play a critical role in semen quality that is associated with male fecundity (Hou et al., 2013; Weng et al., 2014). In addition, semen can be a major vector for the sexual transmission of pathogens including HIV (Liu et al., 2015).

Multiple factors may influence the composition of genital-associated microbiota, including race, age, lifestyle, and sexual activity (Lewis et al., 2017; Noyes et al., 2018). During sexual intercourse, the genital microbiome can be exchanged between sexual partners, and the exchange may have significant influences on the vaginal microbiome, and to a less extent on the semen microbiome (Starnbach and Roan, 2008; Nelson et al., 2012; Liu et al., 2015; Zozaya et al., 2016; Plummer et al., 2018). Mändar et al. (2015) investigated the genital tract microbiota of 23 couples before and after intercourse, and postulated that there was association between semen and vaginal microbiomes. Their study revealed that the seminal microbiome caused the significant decrease in the relative abundance of *Lactobacillus crispatus* after intercourse, and *Gardnerella vaginalis* tend to dominate the vaginal communities of the women whose partners had leukocytospermia (Mändar et al., 2015). Vodstrcil et al.’s (2017) longitudinal sampling of the vaginal microbiome of 52 young women also revealed that penile-vaginal sex changed the vaginal communities into the *Gardnerella vaginalis* dominated microbiome. In spite of these apparently dramatic changes that occurred in vaginal microbiome after sexual intercourse, the relatively long term effects of the intercourse may be limited because of the resilience of normal vaginal microbiota (Borovkova et al., 2011). In addition, the evolutionary implications of the microbiome transmission *via* sexual intercourse are still little known (Ma and Taylor, 2020).

Existing literature on the influence of sexual intercourse on vaginal microbiome clearly highlights its healthy implications for woman (Starnbach and Roan, 2008; Ma et al., 2012; Nelson et al., 2012; Liu et al., 2015; Zozaya et al., 2016; Plummer et al., 2018). Nevertheless, existing studies ignored one important aspect, i.e., what is the microbial transmission (transfer) mechanism during the sexual intercourse? Is it stochastic, passive diffusion similar to the random walk of particles in physics, or driven by some deterministic forces? Is it possible to get rational estimation of the transmission probability and/or the portion of transmitted microbes? Indeed, it may not be practical to obtain such quantifications through experimental or observational studies. Fortunately, it is possible to get rational estimation for such important parameters through mathematical analysis based on the neutral theory of biodiversity (Hubbell, 2001; Li and Ma, 2016; Harris et al., 2017; Ma and Li, 2019; Ma, 2020, 2021a,b). In the present study, we apply Hubbell’s (2001) unified neutral theory of biodiversity (UNTB), specifically the multi-site neutral model (MSN) implemented by Harris et al. (2017) to address the previously identified questions. The neutral theory enables us to determine whether or not the transmission of bacteria during the intercourse is a stochastic event similar to random walk of particles in physics or it is simply deterministic. It also allows for us to get rational estimation for the transmission probability and transmission level. We applied the neutral theory modeling by reanalyzing the microbiome (16s-rRNA) datasets of 23 couples originally reported by Mändar et al. (2015), which constitutes the first objective of the present study—investigating the mechanism of microbiome transmission during the sexual intercourse.

A secondary objective of the present study is to explore the evolutionary implications of the microbiome transmission during the sexual intercourse, which has been rarely addressed in existing literature. For example, one may wonder what are their potential evolutionary implications to the sexual reproduction? Specifically, would the microbiome exchange raise or lower the fitness of sexual reproduction? Indeed, one of the major mysteries of evolutionary biology is why costly sexual reproduction is evolved and maintained, whereas the apparently high efficiency of asexual reproduction is also compelling. That is, why and how would sexual reproduction still be evolved in organisms given the apparently compelling advantage of asexual reproduction, and the mystery has been known as sexual selection problem in literature. The Red Queen hypothesis (Van Valen, 1973; Žliobaitė et al., 2017; Scoville, 2019) has been one of the most favored theories to explain the evolution of sexual reproduction, i.e., a theory for the sexual selection problem. Multiple versions of Red Queen hypothesis have been developed in evolutionary biology. Arguably the most well-known version is the co-evolutionary or arms-race interactions between species (particularly the predator-prey system), in which both the predator and prey must continuously adapt to each other’s innovative, and advantageous mutations to “out-compete” each other, such that neither go extinct and both survive and prosper. According to the Red Queen hypothesis, this arms race or back-and-forth co-evolution of the species is a continuous co-adaptation process over long evolutionary timelines. In the domain of sexual selection, according to the Red Queen theory, sexual reproduction, in which mate can be selected rather than undergoing “closed” and non-selective asexual reproduction, allows for selecting a partner with advantageous characteristics and is therefore more likely to produce offspring better fit for the environment (Scoville, 2019).

In the second mechanism described above for sexual selection (which is followed in this study), it was argued that the evolutionary advantages are particularly strong for one species in a symbiotic relationship if the other species can only undergoes asexual reproduction. For example, since most parasites are asexual, in a host-parasite interaction, if the host can freely select mates that seem immune to the parasite, then the host species would have an evolutionary advantage since its offspring would be more resistant or immune to the parasite. Of course, this does not imply that the parasite could not co-evolve with hosts because it may still accumulate advantageous genes through other means such as simple DNA mutations (Scoville, 2019). We humans are typical sexual reproduction animals, although modern marriage systems may have exerted social limits to the degree of sexual selection. The Red Queen hypothesis has postulated that sexual selection in humans has played a critical role in shaking off some potentially dangerous microbial pathogens, although one may counter-argue that sexual activities *per se* provides venues for sexually transmitted pathogens. While threats of sexually transmitted pathogens are real, a consensus has been that the reproductive system microbiomes (mostly vaginal and semen microbiomes) are generally predominantly beneficial to human hosts such as suppressing/preventing invasions of opportunistic pathogens and maintaining healthy reproductive tract environment (e.g., right acidity in the human vaginal) (e.g., Ma et al., 2012). Nevertheless, comprehensive examinations of the roles of reproductive system microbiomes in human sexual reproduction from an evolutionary perspective are still missing to the best of knowledge.

**FIGURE 1:**
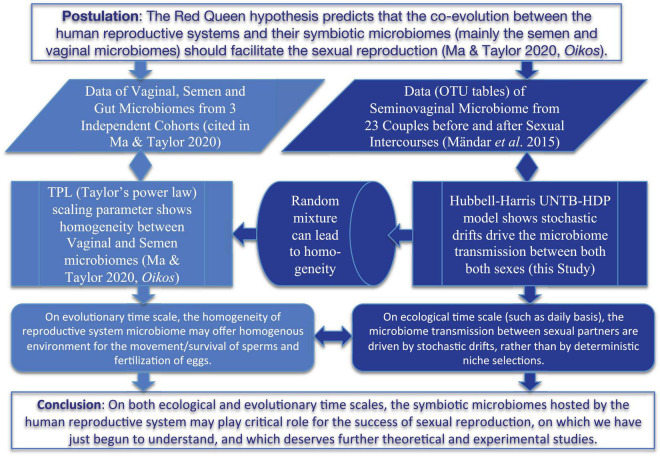
A diagram showing the hypothesis and objectives of this study, including the comparative analysis with previous study (the left side) (Ma and Taylor, 2020).

In a recent study, Ma and Taylor (2020) suggested that co-evolutionary theories such as the Red Queen hypothesis (Van Valen, 1973; Žliobaitė et al., 2017) should be applicable for the co-evolution between human reproductive systems and their symbiotic microbiomes (mainly semen and vaginal microbiomes) due to the microbiome exchanges between both sexes. They argued that the long-term co-evolution should promote the dynamic homogeneity or stability of the microbiomes, possibly being beneficial for sexual reproduction (sexual selection) such as sperm movement and survival as well as egg fertilization. They further tested the hypothesis by analyzing the heterogeneity of the reproductive system (semen and vaginal microbiomes) based on Taylor’s power law (TPL) (Taylor, 1961, 2019) and found no statistically significant differences between the semen and vaginal microbiomes, while both exhibiting significant differences with human gut microbiomes. That is, they demonstrated homogeneity between semen and vaginal microbiomes and therefore indirectly supported the extension of the Red Queen hypothesis to the human reproductive system microbiomes. Nevertheless, the microbiome datasets they used were from independent cohorts, which means that no apparent microbiome exchanges between the men and women in the cohorts actually occurred on ecological time scale. In other words, their results and inferences were on the evolutionary time scale, rather than on the ecological time scale (daily basis). Furthermore, their study only verified the homogeneity but without offering a mechanistic interpretation for the process maintaining the homogeneity at ecological time scale. In the present study, we aim to provide additional evidence to support the Red Queen hypothesis extension to the field of reproductive system microbiomes (Ma and Taylor, 2020) by leveraging the findings from pursuing the previously stated first objective. Specifically, we explore how the mechanism of microbiome transmission during the sexual intercourse influences the heterogeneity (the other side of homogeneity “coin”) of the reproductive system microbiomes on ecological time scale. We conjecture that microbiome transmission during sexual intercourse should promote the homogeneity between semen and vaginal microbiomes on the ecological time scale, similar to what occurs on the evolutionary time scale as suggested by Ma and Taylor (2020). If the conjecture is confirmed, then one may infer that the microbiome transmissions between men and women either through sexual intercourse on ecological time scale or through other means on evolutionary time scale all support the Red Queen hypothesis, namely, that the co-evolution between reproductive system microbiomes and hosts facilitates the sexual reproduction (sexual selection). Figure 1 below diagrammed the hypotheses (objectives) and supporting approaches of the present study. It should be noted that the secondary objective we pursue regarding the Red Queen hypothesis is of conjectural nature since our evidence is indirect and non-experimental. Future studies are required to cross-verify our conjecture.

## Materials and Methods

### Datasets of Microbiome Transmission *via* Sexual Intercourse

Mändar et al. (2015)’s datasets in the form of OTU (operational taxonomic unit) tables, which are reanalyzed in this study, include 23 couples who sought consultation from a physician due to infertility of diverse etiologies. Semen samples were collected by masturbation, and each male was sampled only once. Each female participant was sampled twice, and the vaginal samples were collected in the evening before intercourse and next morning after intercourse. Seminal and vaginal samples were sequenced with Illumina HiSeq2000, and the obtained reads were processed with Mothur software pipeline. A total of 176,358 sequences were obtained, with an average 2,854 reads for each of the 46 vaginal fluid samples, and an average of 1,712 reads for each of the 23 semen samples. Those samples (3 samples per couple, and a total of 69 samples) were ideal materials for investigating microbiome transmission *via* sex, and we take advantage of the neutral theory of biodiversity for determining and estimating the transmission mode and level of the transmission during intercourse. For further information on the cohort information, readers are referred to Mändar et al. (2015). In this study, we used the OTU tables generously supplied to us by the original authors of Mändar et al. (2015).

As a side note, since no second semen samples were taken from the cohort, any discussion on microbiome transmission is primarily one way, from male to female, in this study. Nevertheless, for stable partners, the one-time semen samples cannot exclude the effect of female-to-male transmission obviously.

### Multi-Site Neutral Model Approximated by Hierarchical Dirichlet Process

Hubbell’s (2001) UNTB (unified neutral theory of biodiversity and biogeography) assumes that all individuals from different species are “neutral” in the sense that their differences, even if exist, would not translate into differences in their probabilities of being, and persisting, in the present and future community (Alonso et al., 2006). The neutral theory diametrically contradicts the assumption of classic niche theory, which assumes that different species occupying different niches in their habitats are selected by natural selection to possess different characteristics.

Hubbell’s (2001) UNTB conceptually distinguishes the local community dynamics from meta-community dynamics, but both are driven by similar neutral processes. Meta-community dynamics is controlled by two quantities: speciation probability and reproduction rate of an individual. The diversity of the local community is then maintained by immigration from the meta-community, but no speciation is assumed to occur unlike in the meta-community. With all these assumptions, Hubbell’s neutral theory was formulated as a master equation (a stochastic differential equation), the solution of which is a probability distribution (sampling formula), which can be compared against the species abundance distribution obtained by sampling ecological communities, *via* rigorous statistical testing such as goodness-of-fitting test with χ^2^ statistic.

A fully generalized case of fitting multiple sites UNTB with variable immigration rates among sites is computationally extremely challenging (actually intractable) even for small number of sites, and approximate algorithms must be utilized (Harris et al., 2017). Harris et al. (2017) developed an efficient Bayesian fitting framework by approximating the neutral models with the hierarchical Dirichlet process (HDP). Harris et al. (2017) approximation captures the essential elements of the UNTB, i.e., neutrality, finite populations, and multiple panmictic geographically isolated populations linked by relatively rare migration. With Harris et al.’s (2017) HDP-MSN model, i.e., multi-site neutral (MSN) model approximated by the HDP process, it is possible to simultaneously estimate the variable immigrations rates among a large number of sites within feasible computation timeframe, and therefore makes the UNTB truly multi-site. For this reason, we term Harris et al. (2017) implementation of Hubbell’s UNTB as HDP-MSN model (hierarchical Dirichlet process approximation of multisite neutral model). Furthermore, the HDP-MSN model distinguishes between neutral local community (given a non-neutral meta-community) and the full UNTB (where the meta-community also assembles neutrally), and the neutrality tests can be performed at both meta-community level and local community level.

#### The Unified Neutral Theory of Biodiversity Model

As stated previously, a primary assumption in Hubbell’s UNTB is that both local community dynamics and regional metacommunity dynamics are driven by similar neutral processes, although they are separated conceptually (Hubbell, 2001, 2006). Regarding the local community dynamics, assume there are *M* local communities indexed as *i* = 1, 2… *M*, each with *N*_*i*_ individuals and *N*_*i*_ is constant for each local community. At each time step, the local community dynamics for site *i* is driven by a random process—selecting an individual randomly and either replacing it by a randomly chosen individual immigrated from the metacommunity with migration probability (*m*_*i*_) or replacing it by an indigenous member randomly chosen from the local community (*i*) with probability (1-*m*_*i*_). The UNTB further assumes that the local communities are at stationary state, and each site is assigned a vector ${\bar{\pi }}_{i}$ = (π_*i*,1_, …, π_*i*,*S*_), denoting the probability for observing a particular species at site *i*, which is simply the species abundance distribution (SAD) of site or local community *i*.

One parameter, *immigration rate* (*I*_*i*_), controls the coupling of a local community to the meta-community by replacing the two parameters (*m*_*i*_ and *N*_*i*_), i.e.,


(1) $$I_{i}=\left(N_{i}-1\right)\left[m_{i}/\left(1-m_{i}\right)\right].$$

Regarding the equivalent neutral dynamics of metacommunity, new species are generated through speciation with a probability ν. Similar to local community neutral dynamics, the speciation rate, also known as fundamental biodiversity number (θ), can be defined as:


(2) θ=(ν/(1-ν))(N-1),

where *N* is the fixed (total) number of individuals in the metacommunity. The parameter θ can be considered as the rate at which new individuals are added to the metacommunity as a result of speciation.

The third aspect of the UNTB is to treat the observed samples, i.e., the rows in the data matrix **X**_*MxS*_ with elements *x*_*ij*_ giving the abundance of species *j* is observed at site *i*, as a sample from the local community. As a side note, the matrix X is actually the OTU table of 16s-rRNA gene abundances in the case of test datasets we used in this study. Assume that the sample is taken with replacement, let $J_{i}=\sum_{j=1}^{S}x_{ij}$, and then the multinomial (MN) distribution describes the vector of observations at a given site *i*, i.e.,


(3) $${\bar{X}}_{i}∼MN\left(J_{i},{\bar{\pi }}_{i}\right).$$

In summary, the above three elements (the immigration rate, speciation rate, and multinomial distribution) constitute the building blocks of the neutral theory. These building blocks, together with the neutrality assumption—that all individuals from different species are “neutral” in the sense that their differences, even if exist, would not translate into differences in their probabilities of being, and persisting, in the present and future community (Alonso et al., 2006), may be implemented slightly differently in the following multi-site neutral (MSN) model by Harris et al. (2017). However, the fundamental ideas and elements of neutral theory are the same with classic neutral theory.

#### Hierarchical Dirichlet Process-Multi-Site Neutral Model

Neutral theory is one of the four paradigms of metacommunity theory. Since metacommunity consists of multiple local communities, it is essentially a multi-site model. It turned out that a fully general case of fitting multiple sites UNTB with different immigration rates is computationally extremely challenging (actually intractable) even for small number of sites, and approximate algorithms must be utilized (Harris et al., 2017). Harris et al. (2017) developed an efficient Bayesian fitting framework by approximating the neutral models with the hierarchical Dirichlet process (HDP). Harris et al. (2017) approximation captures the essential elements of the UNTB, i.e., neutrality, finite populations, and multiple panmictic geographically isolated populations linked by relatively rare migration—while little influenced by the specific details of the local community dynamics.

Sloan et al. (2006, 2007) showed that for large local population sizes, and assuming a fixed finite-dimensional metacommunity distribution with *S* species present, then the local community distribution, ${\bar{\pi }}_{i}$, can be approximated by a Dirichlet distribution (Sloan et al., 2006, 2007). But it was Harris et al. (2017) developed the general framework for approximating the UNTB computationally efficiently. Assuming there is a potentially infinite number of species that can be observed in the local community, then the stationary distribution of observing local population *i* is a Dirichlet process (DP), i.e.,


(4) π¯i|Ii,β¯∼DP(I,iβ¯)

where $\bar{\beta }=\left(\beta_{1},\ldots ,\beta_{S}\right)$ is the *relative frequency* of each species in the metacommunity.

At the metacommunity level, a Dirichlet process is still applicable, but then the base distribution is simply a uniform distribution over arbitrary species labels, and the concentration parameter is the biodiversity parameter (θ) (Harris et al., 2017). The metacommunity distribution follows the stick breaking process, i.e.,


(5) $$\bar{\beta }∼Stick\left(\theta \right).$$

Given that both local community and metacommunity are Dirichlet processes, it becomes a hierarchical Dirichlet process (HDP) in terms of the machine learning (Teh et al., 2006; Harris et al., 2017).

Alternatively, Dirichlet process can also be viewed as the so-termed Chinese restaurant process, from which the Antoniak equation can be derived. Antoniak equation represents the number of types (or species) (*S*) observed following *N* draws from a DP with concentration parameter θ and is in the following form:


(6) $$P\left(S|\theta ,N\right)=s\left(N,S\right)\theta^{S}\frac{\Gamma \left(\theta \right)}{\Gamma \left(\theta +N\right)}$$

where *s*(*N*, *S*) is the unsigned Stirling number of the first kind and Γ(.) denotes the gamma function (Antoniak, 1974).

#### Gibbs Sampler (MCMC Algorithm) for the Hierarchical Dirichlet Process-Multi-Site Neutral Model

The full HDP approximated neutral model (HDP-neutral) is formed by combining previous equations (4–6). Harris et al. (2017) devised an efficient Gibbs sampler for the HDP neutral approximation, which is a type of Bayesian Markov Chain Monte Carlo (MCMC) algorithm and can be summarized as the following four sampling steps:

(*a*) Sample the biodiversity paramete*r* (θ) from the conditional probability:


(7) $$P\left(\theta |S,T\right)\propto s\left(T,S\right)\theta^{S}\frac{\Gamma \left(\theta \right)}{\Gamma \left(\theta +T\right)}Gamma\left(\theta |\alpha ,\zeta \right)$$

where θ is the biodiversity parameter. $T=\sum_{i=1}^{M}\sum_{j=1}^{S}T_{ij}$ is the number of ancestors, *S* is the number of species in metacommunity, *s*(*T*, *S*) is the unsigned Stirling number of the first kind (Antoniak, 1974), and α and ζ are constants.

(*b*) Sample the metacommunity distribution:


(8) $$\bar{\beta }=\left(\beta_{1},\beta_{2}\ldots ,\beta_{S},\beta_{u}\right)∼DP\left(T_{\cdot 1},T_{\cdot 2},\ldots ,T_{\cdot S},\theta \right)$$

where $T_{\cdot j}=\sum_{i=1}^{M}T_{ij}$ is the number of ancestors of species *j* in metacommunity.

(*c*) Sample the immigration rates:


(9) P(Ii|T)ij∝Γ(Ii)Γ(Ji+Ii)IiTiGamma(Ii|η,ν)

where both η and ν are constants.

(*d*) Sample the ancestral states:


(10) $$P\left(T_{ij}|x_{ij},I_{i},\beta_{i}\right)=\frac{\Gamma \left(I_{i}\beta_{j}\right)}{\Gamma \left(x_{ij}+I_{i}\beta_{j}\right)}s\left(x_{ij},T_{ij}\right){\left(I_{i}\beta_{j}\right)}^{T_{ij}}$$

where the various symbols have the same representations as previously defined.

Harris et al. (2017) discovered through experiments that to ensure sampling was from the stationary distribution, 50,000 Gibb samples for each fitted dataset were required with the first 25,000 iterations removed as burn-in. The results are reported as the *median* values over the last 25,000 samples with upper and lower credible limits (Bayesian confidence) given by 2.5% and 97.5% quantiles of those samples.

#### Goodness-of-Fitting Test for the Hierarchical Dirichlet Process-Multi-Site Neutral Model

To determine whether an observed dataset fits to the HDP-neutral model, Harris et al. (2017) proposed a similar Monte Carlo significance test to that used by Etienne (2007). For both the local and metacommunity level tests, samples were generated from 2,500 sets of fitted parameters, which were in turn sampled from every tenth iteration of the last 25,000 Gibbs samples (the first 25,000 were removed as burn-in as mentioned previously). The calculation and usage of the pseudo-*P* values for testing the goodness-of-fitting of the HDP-neutral model are explained in the footage for Table 1 in the section of results, where actual model fittings to the datasets of seminovaginal microbiomes are presented. For the detailed computational procedures and computational program, readers are referred to Harris et al. (2017), which we used to perform the microbiome data analysis in this study. In addition, demonstration on the application of HDP-MSN model to the human microbiomes can be found in Ma (2020, 2021a,b).

**TABLE 1:** Test results of fitting the HDP-MSN (hierarchical Dirichlet process, multi-site neutral) model of Harris et al. (2017) to the meta-communities consisting of 3-site semen-vaginal samples (CM = Semen Sample, CNA = vaginal sample before intercourse, and CNB = vaginal sample after intercourse) (*P-*value > 0.05 indicating significant or satisfactory fitting to the MSN)*,**.

ID	*L*_*O*_	θ	*m*	*M-value*	Meta-community				Local Community			
					*L_M_*	*N*_*M*_	*N*	*P*_*M*_	*L_L_*	*N*_*L*_	*N*	*P*_*L*_
1	–382.709	40.978	0.151	73.153	–396.822	1,566	2,500	0.626	–389.420	1,417	2,500	0.567
2***	−1, 385.486	141.111	0.015	37.188	−1, 370.380	1,073	2,500	0.429	−1, 415.622	1,632	2,500	0.653
3	–708.010	91.888	0.013	35.028	–727.026	1,551	2,500	0.620	–744.482	1,775	2,500	0.710
6	−1, 032.545	100.840	0.020	39.051	−1, 051.866	1,499	2,500	0.600	−1, 070.271	1,803	2,500	0.721
7	−1, 040.569	97.987	0.010	32.693	−1, 069.604	1,594	2,500	0.638	−1, 091.907	1,930	2,500	0.772
8	–753.034	73.124	0.029	59.935	–837.017	2,162	2,500	0.865	–804.300	1,990	2,500	0.796
9	–820.546	106.933	0.012	24.899	–835.507	1,468	2,500	0.587	–855.005	1,776	2,500	0.710
12	–425.321	47.271	0.022	43.152	–460.434	1,819	2,500	0.728	–464.377	1,971	2,500	0.788
14	–717.459	93.516	0.011	33.766	–725.231	1,374	2,500	0.550	–742.193	1,638	2,500	0.655
15	–425.352	40.732	0.040	104.209	–476.039	2,024	2,500	0.810	–448.788	1,722	2,500	0.689
16	–692.768	95.069	0.010	27.169	–730.169	1,788	2,500	0.715	–732.498	1,800	2,500	0.720
17	–874.131	102.123	0.007	18.343	–917.696	1,813	2,500	0.725	–949.243	2,146	2,500	0.858
18	–768.402	97.810	0.011	26.005	–808.089	1,771	2,500	0.708	–819.856	1,986	2,500	0.794
21	−1, 381.209	129.878	0.021	40.338	−1, 392.766	1,381	2,500	0.552	−1, 431.878	1,936	2,500	0.774
22	–690.534	70.839	0.015	40.315	–739.996	1,910	2,500	0.764	–741.027	2,012	2,500	0.805
23	−1, 044.392	49.574	0.041	105.839	−1, 277.726	2,478	2,500	0.991	−1, 099.484	2,078	2,500	0.831
24	–740.746	47.534	0.072	151.599	–880.842	2,428	2,500	0.971	–762.288	1,649	2,500	0.660
25	−1, 262.028	79.649	0.024	76.674	−1, 386.549	2,225	2,500	0.890	−1, 301.471	1,725	2,500	0.690
26	−1, 178.871	96.351	0.016	38.733	−1, 290.164	2,253	2,500	0.901	−1, 256.236	2,161	2,500	0.864
27	–998.888	55.951	0.056	160.489	−1, 127.655	2,303	2,500	0.921	−1, 000.514	1,277	2,500	0.511
28	−1, 059.024	62.918	0.076	207.492	−1, 185.529	2,296	2,,500	0.918	−1, 035.615	820	2,500	0.328
29	−1, 086.036	60.456	0.040	126.032	−1, 339.392	2,490	2,500	0.996	−1, 118.532	1,749	2,500	0.700
30	–981.639	66.499	0.013	35.685	−1, 081.354	2,207	2,500	0.883	−1, 055.134	2,176	2,500	0.870
Mean	–889.117	80.393	0.032	66.860	–961.211	1,890	2,500	0.756	–927.397	1,790	2,500	0.716
Passing rate (%)								100%				100%

*N = 2,500 is the number of Gibb samples selected from 25,000 simulated communities (i.e., every tenth iteration of the last 25,000 Gibbs samples), it is chosen to compute the pseudo P-value below for conducting the neutrality test.

*L_0_ is the actual (observed) log-likelihood.*

*θ is the median of biodiversity numbers computed from 25,000 times of simulations.*

*m is the migration probability.*

*M-value is the average medians of the migration rates of local communities in each meta-community (i.e., the average median of the individuals migrated per generation), also computed from 25,000 times of simulations.*

*L_M_ is the median of the log-likelihoods of the simulated neutral meta-community samples; and N_M_ is the number of simulated neutral meta-community samples with their likelihoods satisfying L_M_ ≤ L_0_ (where L_M_ and L_0_ are the simulated and actual likelihood respectively).*

*P_M_ = N_M_ /N is the pseudo p-value for testing the neutrality at meta-community level; if P_M_ > 0.05, the meta-community is indistinguishable from the prediction of neutral model.*

*L_L_ is the median of the log-likelihoods of the simulated local community samples, and N_L_ is the number of simulated local community samples with their likelihoods satisfying L_L_ ≤ L_0_ (where L_L_ and L_0_ are the simulated and actual likelihood respectively).*

*P_L_ = N_L_ /N, is the pseudo p-value for testing the neutrality at the local community level; if P_L_ > 0.05, the local community satisfies the neutral model.*

**Due to the typo/error in Harris et al. (2017), the P_M_-values exhibited here are adjusted as (P_M_ = 1−P_MS_), where P_MS_ is output from their computational program.

**FIGURE 2:**
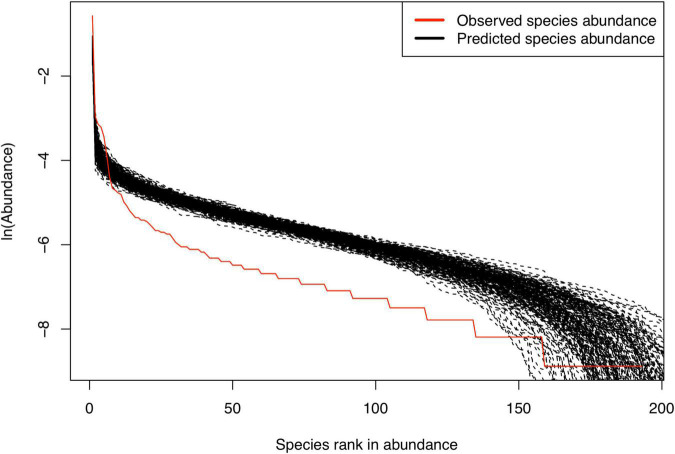
Fitting Harris et al. (2017) MSN (multi-site neutral) model with the meta-community of (CM+CNA+CNB) samples from a randomly selected sample group (Couple#2).

Similarly, the P_L_-values are adjusted as (P_L_ = 1−P_LS_), where P_LS_ is output from their computational program. ***Figure 2 displayed the fitting of the MSN to #2 sample by plotting the predicted and observed species abundance rank distribution.

## Results and Conclusion

Table 1 listed the test results of fitting the MSN (multi-site neutral) model of Harris et al. (2017) to the semen-vaginal meta-community, which consists of the three samples from each couple (i.e., CM = semen sample, CNA = vaginal sample before intercourse, and CNB = vaginal sample after intercourse). A total of 23 meta-communities, one for each couple, were tested for their fitness to the MSN model, respectively. The neutrality-passing rate in the 23 couples is 100% (all 23) both at the local community and metacommunity level (Figure 2). Table 2 listed the test results of fitting the MSN model, with pair of samples from a couple grouped as a meta-community. There are three possible pair-wise combinations, CM and CNA, CM and CNB, and CNA and CNB. The meta-communities from all pairs passed the neutrality test at both local and meta-community level (100% passing rates). These findings suggest that the transmission of microbes during sexual intercourse seems to be similar to a random “walk” (or random dispersal) and is driven by stochastic drifts. The 100% passing rate indicates that deterministic selection (forces) seems to play little role. Table 2 also suggested that the transmission probability of microbiomes through sexual intercourse appears to be 0.05 approximately.

**TABLE 2:** Test results of fitting the HDP-MSN (hierarchical Dirichlet process, multi-site neutral) model of Harris et al. (2017) to the 2-sites meta-community (pair-wise combination of CM, CNA, CNB) (*p* > 0.05 indicating significant or satisfactory fitting to the MSN)*.

ID	*L*_*O*_	θ	*m*	*M-value*	Meta-community				Local Community			
					*L_M_*	*N*_*M*_	*N*	*P*_*M*_	*L_L_*	*N*_*L*_	*N*	*P*_*L*_
**Meta-community = CM (semen) and CNA (vaginal before): i.e., Semen-Vaginal before Sex**												
1	–294.942	38.974	0.269	127.603	–281.606	891	2,500	0.356	–281.056	827	2,500	0.331
2	−1, 249.588	167.832	0.019	48.708	−1, 178.198	477	2,500	0.191	−1, 234.428	1,016	2,500	0.406
3	–606.843	104.394	0.018	46.314	–586.250	930	2,500	0.372	–607.972	1,267	2,500	0.507
6	–941.873	117.855	0.025	48.872	–912.698	859	2,500	0.344	–950.423	1,395	2,500	0.558
7	–641.123	113.683	0.013	34.910	–613.257	792	2,500	0.317	–636.724	1,162	2,500	0.465
8	–519.786	56.211	0.145	185.879	–545.308	1,648	2,500	0.659	–521.949	1,292	2,500	0.517
9	–557.682	126.973	0.030	29.291	–523.500	701	2,500	0.280	–545.089	1,039	2,500	0.416
12	–318.186	47.318	0.242	67.048	–316.137	1,196	2,500	0.478	–323.589	1,367	2,500	0.547
14	–637.346	139.845	0.009	33.859	–591.837	599	2,500	0.240	–619.023	975	2,500	0.390
15	–445.940	80.545	0.044	35.486	–426.659	926	2,500	0.370	–432.225	1,072	2,500	0.429
16	–602.985	151.693	0.011	26.547	–564.504	752	2,500	0.301	–590.195	1,061	2,500	0.424
17	–722.901	154.230	0.007	20.553	–716.408	1,150	2,500	0.460	–750.418	1,676	2,500	0.670
18	–649.086	103.733	0.015	35.366	–639.784	1,090	2,500	0.436	–658.020	1,420	2,500	0.568
21	−1, 041.511	162.229	0.019	41.977	−1, 007.968	791	2,500	0.316	−1, 050.771	1,406	2,500	0.562
22	–565.380	85.202	0.016	46.302	–571.889	1,353	2,500	0.541	–584.253	1,574	2,500	0.630
23	–687.356	49.269	0.066	139.642	–778.801	2,240	2,500	0.896	–682.369	1,149	2,500	0.460
24	–463.632	39.916	0.229	384.019	–514.220	2,094	2,500	0.838	–455.452	998	2,500	0.399
25	−1, 050.043	89.466	0.023	92.515	−1, 095.046	1,699	2,500	0.680	−1, 053.375	1,268	2,500	0.507
26	–977.067	152.656	0.011	31.491	–973.298	1,189	2,500	0.476	−1, 002.310	1,629	2,500	0.652
27	–886.104	75.389	0.029	99.172	–932.443	1,647	2,500	0.659	–886.128	1,251	2,500	0.500
28	–838.843	60.589	0.103	335.688	–875.010	1,775	2,500	0.710	–790.556	342	2,500	0.137
29	−1, 029.231	114.178	0.014	51.628	−1, 097.116	1,962	2,500	0.785	−1, 068.638	1,509	2,500	0.604
30	–837.386	105.981	0.009	28.098	–857.412	1,513	2,500	0.605	–878.143	1,861	2,500	0.744
Mean	–720.210	101.659	0.059	86.564	–721.711	1,229	2,500	0.492	–721.874	1,242	2,500	0.497
Passing rate (%)								100%				100%
**Meta-community = CM (semen) and CNB (vaginal after): i.e., Semen-Vaginal after Sex**												
1	–289.030	43.371	0.204	105.245	–280.867	1,056	2,500	0.422	–282.879	1,056	2,500	0.426
2	–727.836	129.926	0.024	39.766	–688.822	677	2,500	0.271	–718.518	677	2,500	0.442
3	–511.738	66.266	0.054	99.539	–493.772	958	2,500	0.383	–498.098	958	2,500	0.418
6	–524.047	62.040	0.104	109.920	–516.595	1,127	2,500	0.451	–513.470	1,127	2,500	0.437
7	–922.580	120.417	0.016	41.932	–900.271	950	2,500	0.380	–936.888	950	2,500	0.585
8	–664.375	80.807	0.036	74.639	–683.925	1,547	2,500	0.619	–680.023	1,547	2,500	0.586
9	–734.546	127.632	0.013	31.013	–707.562	874	2,500	0.350	–740.545	874	2,500	0.537
12	–327.890	66.144	0.022	42.932	–314.591	994	2,500	0.398	–327.296	994	2,500	0.494
14	–619.570	92.639	0.036	51.576	–591.182	810	2,500	0.324	–610.084	810	2,500	0.445
15	–347.050	36.931	0.073	194.679	–366.486	1,617	2,500	0.647	–350.091	1,617	2,500	0.533
16	–657.279	148.840	0.012	26.854	–629.818	838	2,500	0.335	–658.247	838	2,500	0.506
17	–668.278	122.494	0.013	23.030	–666.608	1,219	2,500	0.488	–696.241	1,219	2,500	0.694
18	–660.140	139.453	0.015	27.505	–626.643	757	2,500	0.303	–659.396	757	2,500	0.495
21	–845.976	140.958	0.041	41.885	–799.162	567	2,500	0.227	–836.271	567	2,500	0.428
22	–600.325	102.615	0.022	37.687	–575.632	844	2,500	0.338	–595.463	844	2,500	0.465
23	–739.692	52.565	0.051	126.679	–836.581	2,237	2,500	0.895	–753.233	2,237	2,500	0.594
24	–673.891	54.907	0.055	140.013	–756.745	2,174	2,500	0.870	–684.364	2,174	2,500	0.554
25	−1, 040.640	75.592	0.037	141.517	−1, 102.943	1,898	2,500	0.759	−1, 028.525	1,898	2,500	0.442
26	−1, 041.126	127.860	0.013	41.347	−1, 077.386	1,715	2,500	0.686	−1, 091.201	1,715	2,500	0.759
27	–794.096	49.538	0.115	397.648	–863.984	2,040	2,500	0.816	–767.550	2,040	2,500	0.293
28	–957.051	62.429	0.084	326.120	−1, 042.312	2,170	2,500	0.868	–920.118	2,170	2,500	0.235
29	–860.525	55.146	0.059	231.842	–986.236	2,364	2,500	0.946	–843.578	2,364	2,500	0.364
30	–768.696	64.007	0.020	54.922	–811.856	1,772	2,500	0.709	–791.770	1,772	2,500	0.611
Mean	–694.625	87.938	0.049	104.708	–709.564	1,357	2,500	0.543	–694.950	1,357	2,500	0.493
Passing rate (%)								100%				100%
**Meta-community = CNA + CNB: i.e., two vaginal samples**												
1	–117.932	6.528	0.151	85.064	–146.579	2,029	2,500	0.812	–115.060	1,077	2,500	0.431
2	–687.812	52.987	0.026	80.201	–806.923	2,385	2,500	0.954	–704.006	1,556	2,500	0.622
3	–237.691	36.215	0.001	4.373	–285.973	2,096	2,500	0.838	–288.602	2,197	2,500	0.879
6	–558.172	98.290	0.006	15.714	–571.206	1,444	2,500	0.578	–591.573	1,820	2,500	0.728
7	–495.413	39.212	0.009	38.126	–615.493	2,410	2,500	0.964	–543.052	1,871	2,500	0.748
8	–274.276	51.506	0.002	4.737	–320.860	2,072	2,500	0.829	–327.750	2,184	2,500	0.874
9	–375.537	72.550	0.003	6.641	–388.970	1,514	2,500	0.606	–402.978	1,779	2,500	0.712
12	–211.853	24.140	0.001	3.883	–255.803	2,085	2,500	0.834	–253.221	2,145	2,500	0.858
14	–211.848	46.287	0.001	3.275	–243.275	1,846	2,500	0.738	–247.457	2,038	2,500	0.815
15	–120.950	23.085	0.000	1.592	–144.765	1,868	2,500	0.747	–145.086	1,899	2,500	0.760
16	–221.478	49.669	0.001	3.189	–253.301	1,905	2,500	0.762	–260.003	2,068	2,500	0.827
17	–377.262	34.993	0.003	11.786	–443.105	2,172	2,500	0.869	–418.600	1,968	2,500	0.787
18	–255.349	42.477	0.002	4.744	–300.117	2,090	2,500	0.836	–306.651	2,219	2,500	0.888
21	–940.462	130.900	0.012	29.270	–947.139	1,351	2,500	0.540	–978.608	1,844	2,500	0.738
22	–267.858	38.835	0.001	4.851	–306.574	1,996	2,500	0.798	–316.669	2,160	2,500	0.864
23	–612.796	32.891	0.041	133.344	–782.288	2,465	2,500	0.986	–636.483	1,830	2,500	0.732
24	–352.422	58.629	0.004	8.755	–405.049	2,085	2,500	0.834	–380.030	1,539	2,500	0.616
25	–342.730	19.430	0.049	100.793	–477.573	2,462	2,500	0.985	–356.722	1,688	2,500	0.675
26	–363.988	22.576	0.073	118.692	–488.442	2,457	2,500	0.983	–365.290	1,295	2,500	0.518
27	–270.576	14.159	0.050	94.893	–377.782	2,429	2,500	0.972	–274.642	1,391	2,500	0.556
28	–205.859	12.578	0.126	152.710	–290.304	2,433	2,500	0.973	–202.680	1,105	2,500	0.442
29	–298.507	15.997	0.071	137.058	–420.280	2,451	2,500	0.980	–306.712	1,524	2,500	0.610
30	–322.943	16.873	0.059	123.100	–434.570	2,425	2,500	0.970	–329.146	1,443	2,500	0.577
Mean	–353.205	40.905	0.030	50.730	–422.016	2,107	2,500	0.843	–380.479	1,767	2,500	0.707
Passing rate (%)								100%				100%

*The interpretations of the symbols are the exactly the same as in Table 1.

Hence, our analysis revealed that the microbiome transmission during the intercourse is primarily driven by stochastic neutral drift alone and should just be a random walk. The virtually universal neutrality among all the samples suggest that the neutrality is maintained within couples on daily basis, rather than only during the sexual intercourse. It should also be plausible to conjecture that the neutrality may possess both ecological and evolutionary implications, which we further elaborate in the discussion section.

**FIGURE 3:**
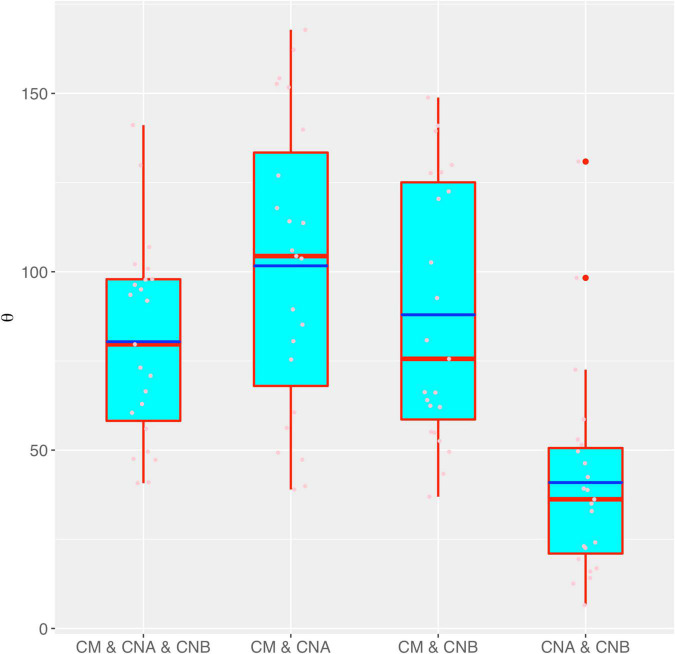
The box chart for the fundamental biodiversity (θ) numbers estimated with the MSN models for the four different meta-community settings (also see Table 3 for the *p*-values of the significance test for their differences in θ). Box red lines, blue lines, edges, whiskers, and bigger red points signify the median, mean, inter-quartile range (IQR), 1.5 × IQR, and > 1.5 × IQR, respectively. The smaller points in each box are the real values of θ of each sample.

Figure 3 shows the box chart for the fundamental biodiversity (θ) numbers estimated with the MSN models for the four different meta-community settings, as listed in Tables 1, 2. It confirms the previous conclusions we draw from the neutrality tests with the MSN modeling reported in Tables 1, 2. Specifically, θ is the lowest in the meta-community of the two vaginal samples setting (CNA and CNB), which simply indicates that the number of “novel” species (regional diversity) in the meta-community of two-sample vaginal microbiome is the lowest, compared with the other three meta-community settings, in which both semen and vaginal communities are included. This should be expected since the “range” of CNA and CNB metacommunity should be smaller than that of the others, and therefore hosts smaller microbiome diversity.

**TABLE 3:** The *p*-value from the Wilcoxon non-parametric test for the immigration probability (*m*) and the fundamental biodiversity number (θ) in Tables 1, 2.

Meta-community I	Meta-community II	*M*	θ
CM and CNA and CNB	CM and CNA	0.575	0.068
CM and CNA and CNB	CM and CNB	0.054	0.617
CM and CNA and CNB	CNA and CNB	0.076	<0.001*
CM and CNA	CM and CNB	0.296	0.326
CM and CNA	CNA and CNB	0.026*	<0.001*
CM and CNB	CNA and CNB	0.012*	<0.001*

*Indicating the treatments with significant difference in the immigration probability at the significance level of P-value = 0.05.

**FIGURE 4:**
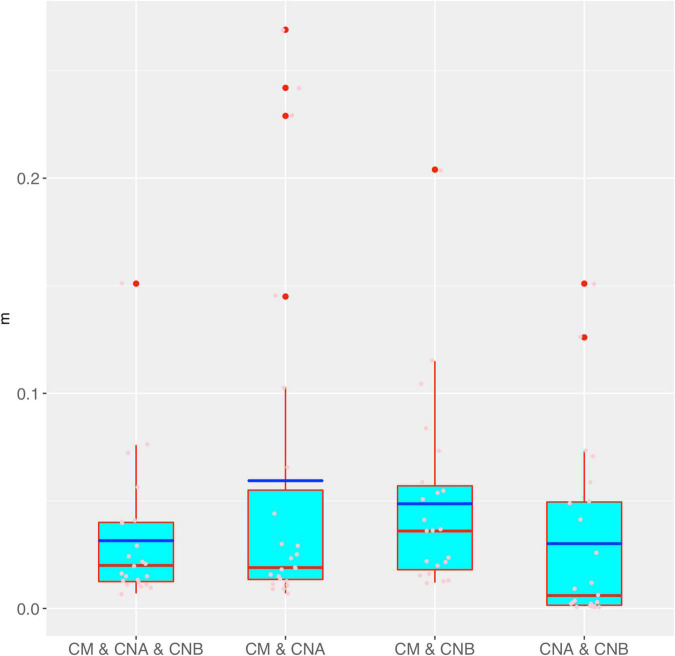
The box chart for the immigration probability (*m*) estimated with the MSN models for the four different meta-community settings (also see Table 3 for the *p*-values of the significance test for their differences in *m*). Box red lines, blue lines, edges, whiskers, and bigger red points signify the median, mean, inter-quartile range (IQR), 1.5 × IQR, and > 1.5 × IQR, respectively. The smaller points in each box are the real values of *m* of each sample.

Figure 4 shows the box chart for the immigration probability (*m*) estimated with the MSN models for the four different meta-community settings, as listed in Tables 1, 2. It confirms the previous conclusion we draw from the MSN neutrality tests reported in Tables 1, 2. Specifically, *m* is the lowest in the meta-community of the two vaginal samples, which simply says that the dispersal (transmission) is the lowest between the two vaginal samples of a woman, compared with the other three meta-communities in which semen microbiome is included. This should be true obviously for the same reason as in the case of previously explained results of *θ.*

**FIGURE 5:**
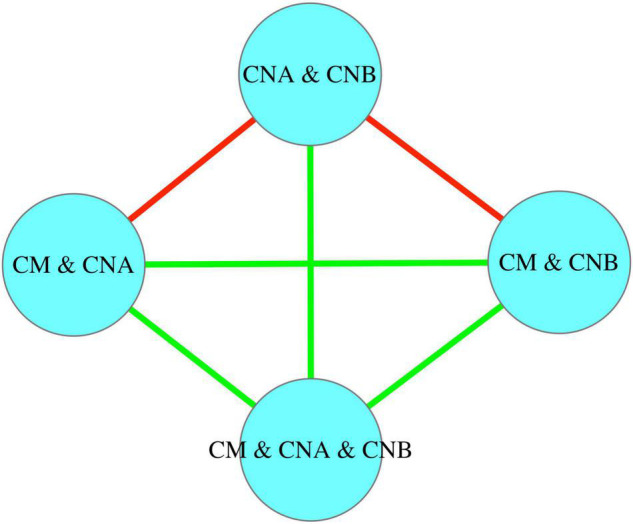
The significance test for the immigration probability (*m*) between different meta-communities: In terms of the immigration probability (*m*), the meta-community of two vaginal samples (“CNA and CNB”) has significant differences (red links) with the meta-communities of “CM and CNA” or “CM and CNB,” and has no significant differences with all other meta-communities (green links).

Table 3 listed the *p*-value from the Wilcoxon non-parametric test for the immigration probability (*m*) and the fundamental biodiversity number (θ). Figure 5 further illustrated the same information as displayed in Table 3. In terms of the immigration probability (*m*), the meta-community of two vaginal samples (CNA and CNB) has significant differences (red links) with the meta-communities of CM and CNA or CM and CNB, and has no significant differences with all other meta-communities (green links). This should be expected, and it simply indicates that the transmission (dispersal) probability between man and woman after intercourse is significantly higher than the immigration probability naturally occurring within the vaginal microbiome.

In summary, previous results have shown that the microbiome transmission during the sexual intercourse appears to be driven by stochastic drifts of microbiome demography and dispersal, rather than by certain deterministic processes such as niche selections (e.g., the preferences of microbes to particular habitats). Further comparisons of the complementary seminovaginal microbiome samples before and after intercourse suggest that the level of stochastic drifts in the semen-vaginal metacommunity should be beyond the duration of sexual intercourse and be predominant on daily basis given that the neutrality passing rates were 100% in both before and after sexual intercourse. In other words, the microbiome exchanges between male and female, at least within couples, on ecological time scale are most likely driven by stochastic drifts and dispersal, rather than by certain deterministic forces.

The previous interpretations of the results are focused on the first or primary objective of this study, i.e., the underlying mechanisms of the microbial community/metacommunity assembly and diversity maintenance, including the possible transmission of microbes during sexual intercourse. As to the secondary objective of this study—the evolutionary implications of the findings of this study—is further discussed in the following section.

## Discussion

There are currently two major categories of hypotheses on the relationship between the evolutions of humans and their symbiotic microbiomes. The emerging theory of evolution considers the individual animal or plant as a community (or a holobiont) consisting of the host plus all of its symbiotic microbes. The collective genome of the holobiont is defined as the hologenome. The holobiont/hologenome theory maintains that the variations in the hologenome can be transmitted from generation to generation with reasonable fidelity, and are subject to evolutionary changes resulting from selection and drift (Rosenberg et al., 2009; Rosenberg and Zilber-Rosenberg, 2018). The theory further maintains that many factors including new acquisitions of microbes, horizontal gene transfers, and changes in microbial species abundance within hosts may cause genetic variation in the hologenome. Due to its mixture flavor of both Lamarckian and Darwinian, the theory stresses both cooperation and competition within and between holobionts (Rosenberg et al., 2009; Rosenberg and Zilber-Rosenberg, 2018), but the overall framework is still Darwinian evolution. The second category emphasizes the co-evolution between the hosts and microbiomes. For example, the classic Red Queen hypotheses (Van Valen, 1973; Žliobaitė et al., 2017) for explaining sexual selection and host/parasite co-evolutions have been applied to interpret the host/microbiome co-evolution (e.g., Papkou et al., 2018; Ma and Taylor, 2020). In reproductive biology, microbial symbionts were found to mediate reproductive isolation in *Drosophila*, but debates also exist (Leftwich et al., 2017; Shapiro, 2017; Schneider et al., 2019). Although, to the best of our knowledge, no experimental studies have been conducted with the human microbiomes, their roles in human reproductive biology cannot be excluded. Theoretically, Ma and Taylor (2020) postulated that the human semen and vaginal microbiomes, collectively termed human reproductive system microbiomes, may have coevolved with hosts to facilitate the sexual reproduction such as offering beneficial environmental for the sperm movement/survival and/or egg fertilization.

While a hallmark of the Red Queen hypothesis is the antagonism or evolutionary conflicts, in which both species are locked in an “arms race” to maximize their relative fitness (Aleru and Barber, 2020), how would the mutualism or evolutionary cooperation between humans and their microbiomes fits to the picture of Red Queen dynamics? In the case of human gut microbiome, it has been found that our immune system is trained to discriminately treat pathogenic bacteria vs. beneficial ones that constitutes majority of the human gut microbiome. Positive selection—the rapid spread of new beneficial gene mutations in populations over time—has been observed in immune system related genes. Indeed, immune system components are among the most rapidly evolving genes in animal genomes. Commensal microbes are believed to be able to shift the balance of host-pathogen conflicts as described by the Red Queen dynamics (Aleru and Barber, 2020). In reproductive biology, microbial symbionts were found to mediate reproductive isolation in *Drosophila*, but debates also exist (Leftwich et al., 2017; Shapiro, 2017; Schneider et al., 2019). It should also be possible that the human and their microbiota have been coevolving with hosts through cooperation, competition (antagonism), and communication (signaling); consequently, the Red Queen type evolutionary dynamics should exist within and between holobiont(s), which are host plus all of its symbiotic microbes (Rosenberg et al., 2009; Rosenberg and Zilber-Rosenberg, 2018).

Ma and Taylor (2020) proposed that the co-evolution between human reproductive system and their symbiotic microbiomes (mainly the semen and vaginal microbiomes) should facilitate the sexual reproduction, as predicted by the classic Red Queen hypothesis. They further provided a piece of evidence to support this microbiome extension to the Red Queen theory by demonstrating that the heterogeneities of semen and vaginal microbiomes exhibited no significant differences, whereas both exhibiting significant differences with human gut microbiomes. Their logic was that the homogeneity or stability should be helpful for the success of sexual reproduction such as being beneficial for the sperm movement/survival and/or egg fertilization. However, Ma and Taylor (2020) study possessed two limitations, as mentioned in previous introduction section, one is the lack of a mechanistic interpretation for why the homogeneity between semen and vaginal microbiomes was the case, and the second is that the microbiome datasets they used were from independent cohorts of men and women (no apparent microbiome exchanges on ecological time-scale such as daily basis), rather than from intimately connected couples as the datasets (Mändar et al., 2015) reanalyzed in this study.

The results from the present study actually fill the two gaps left by Ma and Taylor (2020) study. First, the stochastic drifts or random nature of microbiome exchanges explains the microbiome homogeneity within the reproductive system (i.e., semen and vaginal microbiomes). This is because random migration (mixture) is arguably the most effective mechanism (process) to achieve homogeneity in a fluid environment. Second, the time scale of the reproductive system microbiomes we used in this study is on ecological time scale (daily basis) given that the complementary seminovaginal microbiome samples were obtained both before and after sexual intercourses. Therefore, this study not only offers another piece of evidence to support the prediction of the Red Queen hypothesis on ecological time scale, but also presents a mechanistic interpretation for the process generating the microbiome homogeneity within the reproductive system as revealed by Ma and Taylor’s (2020) previous study, which was postulated on the evolutionary time scale as explained previously. Combined together, both previous study by Ma and Taylor (2020) and the present one seem to confirm that the microbiome transmissions between men and women either through sexual intercourse on ecological time scale or through other means on evolutionary time scale all support the Red Queen hypothesis, namely, that the co-evolution between reproductive system microbiomes and hosts facilitates the sexual reproduction (sexual selection). However, we must reiterate the hypothetic nature of our discussion, that is, all assumptions are subject to further experimental and/or theoretical analyses (verifications).

In summary, this study, integrated with Ma and Taylor (2020), appears to cast relatively complete and reasonably strong evidence to support the extension of the classic Red Queen theory to the field of human reproductive system microbiome. That is, the co-evolution between human reproductive systems and their symbiotic microbiomes should facilitate the sexual reproduction. As the title of the classic monograph “*The Ecological Theater and the Evolutionary Play*” by G. E. Hutchinson (1965), implied, it is the ecology that sets theater (environment background) for evolution (adaptation) to act. We believe that the extension of the classic Red Queen hypothesis to the field of reproductive system microbiomes highlights the critical importance of symbiotic microbes to the success of sexual reproduction, on which our current understanding is still rather limited. Therefore, future theoretic and experimental studies from both ecological and evolutionary perspectives are dearly needed.

Finally, this study possesses several limitations that should be mentioned here. First, the discussion of microbiome transmission is primarily one way from male to female given that only one-time semen sample was taken from each couple in the reanalyzed datasets of Mändar et al. (2015). Second, Mändar et al. (2015) study was originally designed to investigate the relationships between infertility and microbiomes, but the implications of the infertility to the results presented in this reanalysis of their data are unknown due to lack of controls. Third, other factors such as multiple sexual partners, occurrences of diseases such as BV or HIV, are not considered in this study, and their implications are unknown. Fourth, no Type-II error analysis is performed in this study, which could detect false negatives in the neutrality tests or the potential non-neutral processes in those cases that have passed the neutrality test (Ma, 2021a,b). Fifth, as correctly pointed out by an anonymous expert reviewer, Mändar et al. (2015) study used the V6 region, which is not commonly used in vaginal or seminal microbiome studies given its limited ability to resolve gynecological taxa. Furthermore, the database used by Mändar et al. (2015) bioinformatics analysis, i.e., the Greengenes, is somewhat outdated and lacks representatives of understudied niches. Despite these unknown implications, we feel that the findings of this study are very likely robust against most of the additional factors. Part of the somewhat circular arguments comes from the prediction (expectation) of the Red Queen hypothesis. Given these limitations, it should be reiterated that findings in this study should be treated as postulations or evidence to support existing hypotheses (particularly the Red Queen hypothesis). Sometimes, the evidence is indirect or even conjectural, and further experimental and/or theoretical studies are necessary to cross-verify our findings.

## Data Availability Statement

Publicly available datasets were analyzed in this study. The source of the datasets is Mändar et al. (2015).

## Author Contributions

ZM designed the study, interpreted the results, and wrote the manuscript.

## Conflict of Interest

The author declares that the research was conducted in the absence of any commercial or financial relationships that could be construed as a potential conflict of interest.

## Publisher’s Note

All claims expressed in this article are solely those of the authors and do not necessarily represent those of their affiliated organizations, or those of the publisher, the editors and the reviewers. Any product that may be evaluated in this article, or claim that may be made by its manufacturer, is not guaranteed or endorsed by the publisher.

## Abbreviations

BV: bacterial vaginosis
BVAB: BV associated anaerobic bacteria
CM: semen sample
CNA: vaginal sample before intercourse
CNB: vaginal sample after intercourse
HDP: hierarchical Dirichlet process
MSN: multi-site neutral model
HDP-MSN: hierarchical Dirichlet process approximation to multisite neutral model
SAD: species abundance distribution
UNTB: unified neutral theory of biodiversity.
